# Supplementary figures and images for: azyx-1 is a new gene that overlaps with zyxin and affects its translation in C. elegans, impacting muscular integrity and locomotion
Source: PLoS Biol. 2023 Sep 15;21(9):e3002300. doi: 10.1371/journal.pbio.3002300 (PMC10575671; doi:10.1371/journal.pbio.3002300)

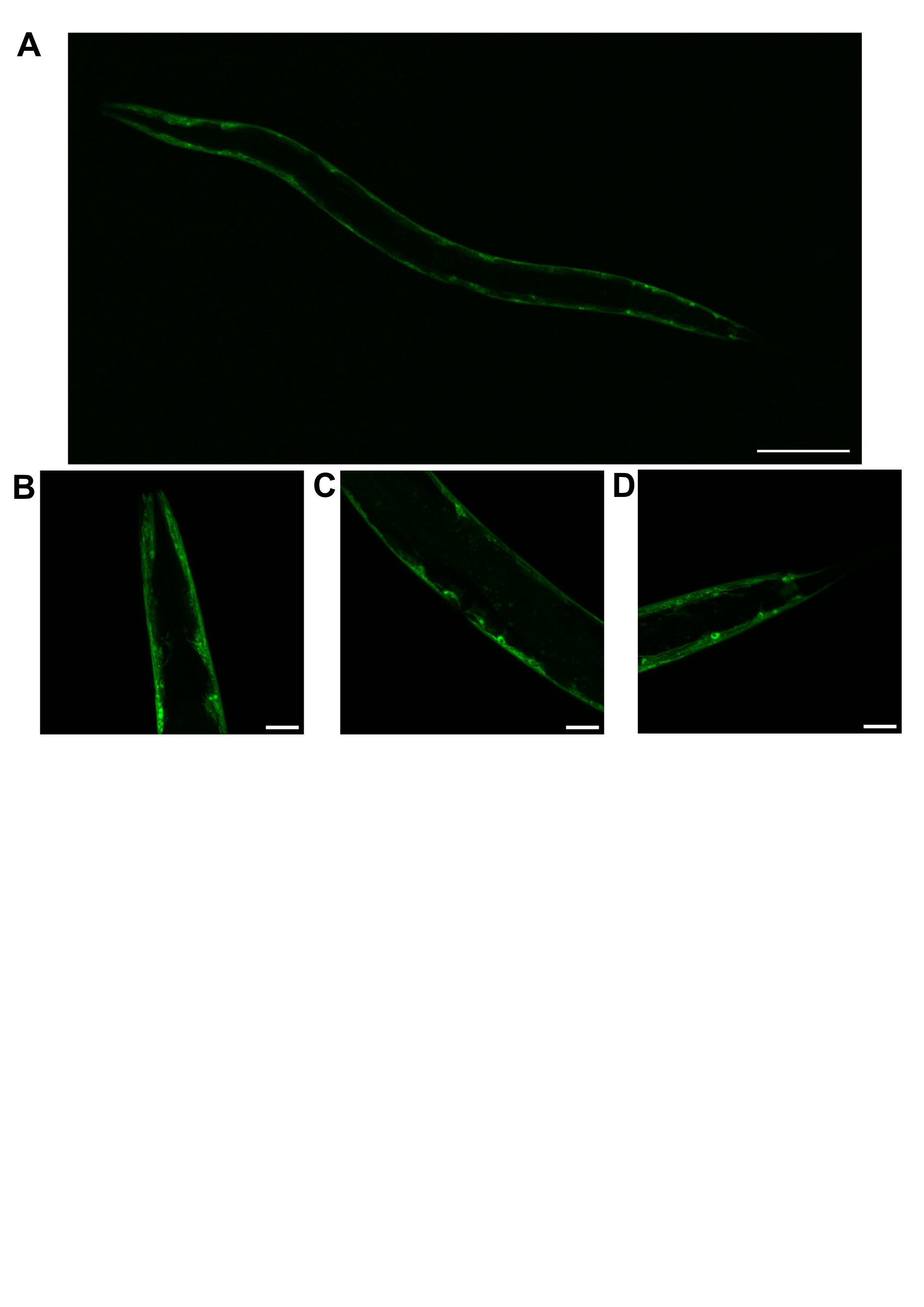

Supplement: S1 Fig — Localization of azyx-1p::azyx-1+mNeonGreen::UTR in (A) body wall (scale bar, 100 μm), (B) head, (C) vulval muscle, and (D) tail (scale bar, 20 μm) of L4 stage worms. (TIF) [file pbio.3002300.s001.tif]

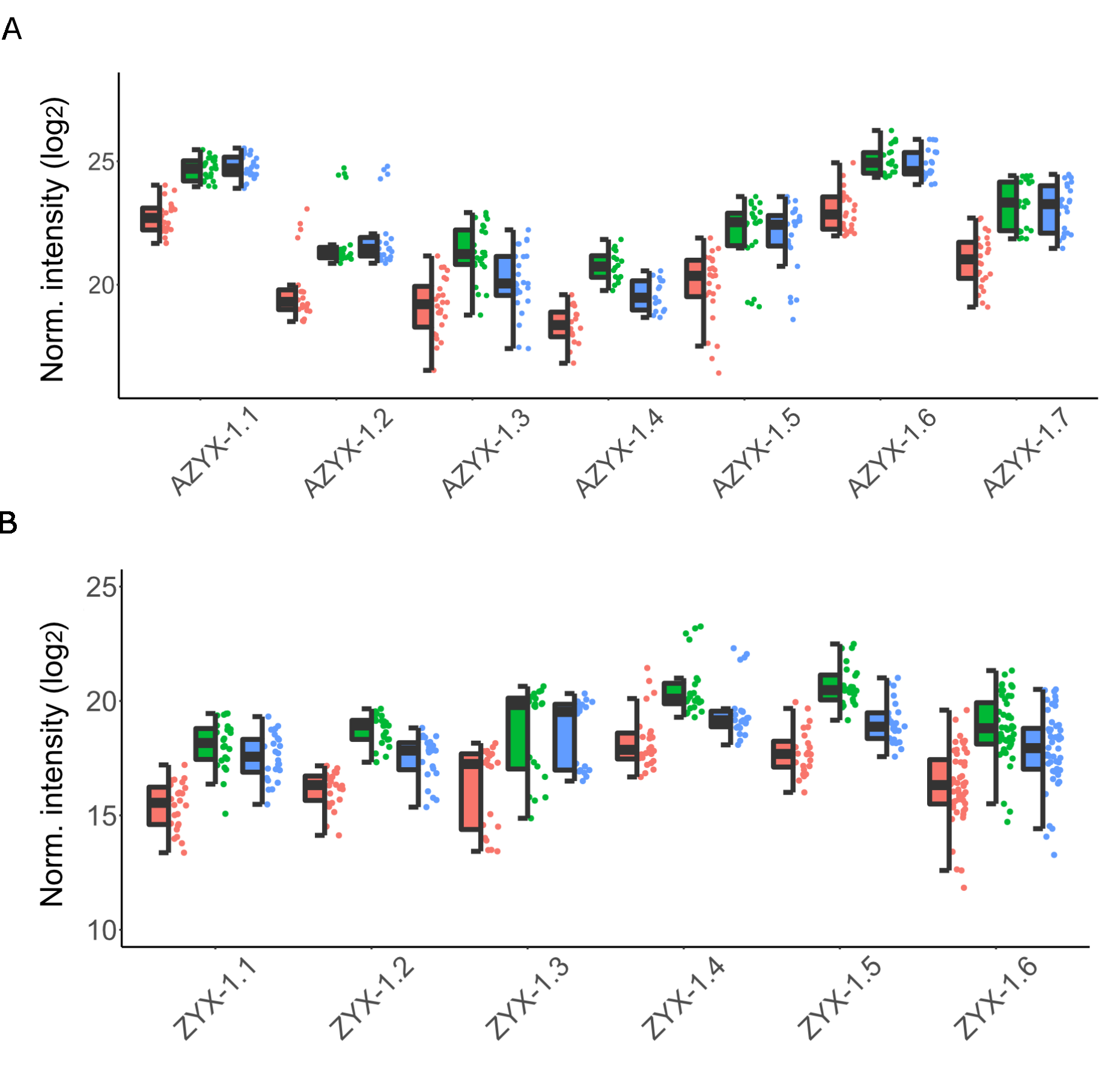

Supplement: S2 Fig — Normalized intensity of transition ions for peptides corresponding to (A) AZYX-1 (7 peptides) and (B) ZYX-1 (6 peptides) measured at L4 (orange), day 1 (green) and day 8 (blue) of adulthood (n = 4) with mean intensity across replicates (horizontal black line). Data normalized to spike-in (1 fmol/worm). Formal statistical analysis of data was performed and indicated in main text corresponding to Fig 3A–3D. Data used to generate figures can be found in S1 Data. (TIF) [file pbio.3002300.s002.tif]

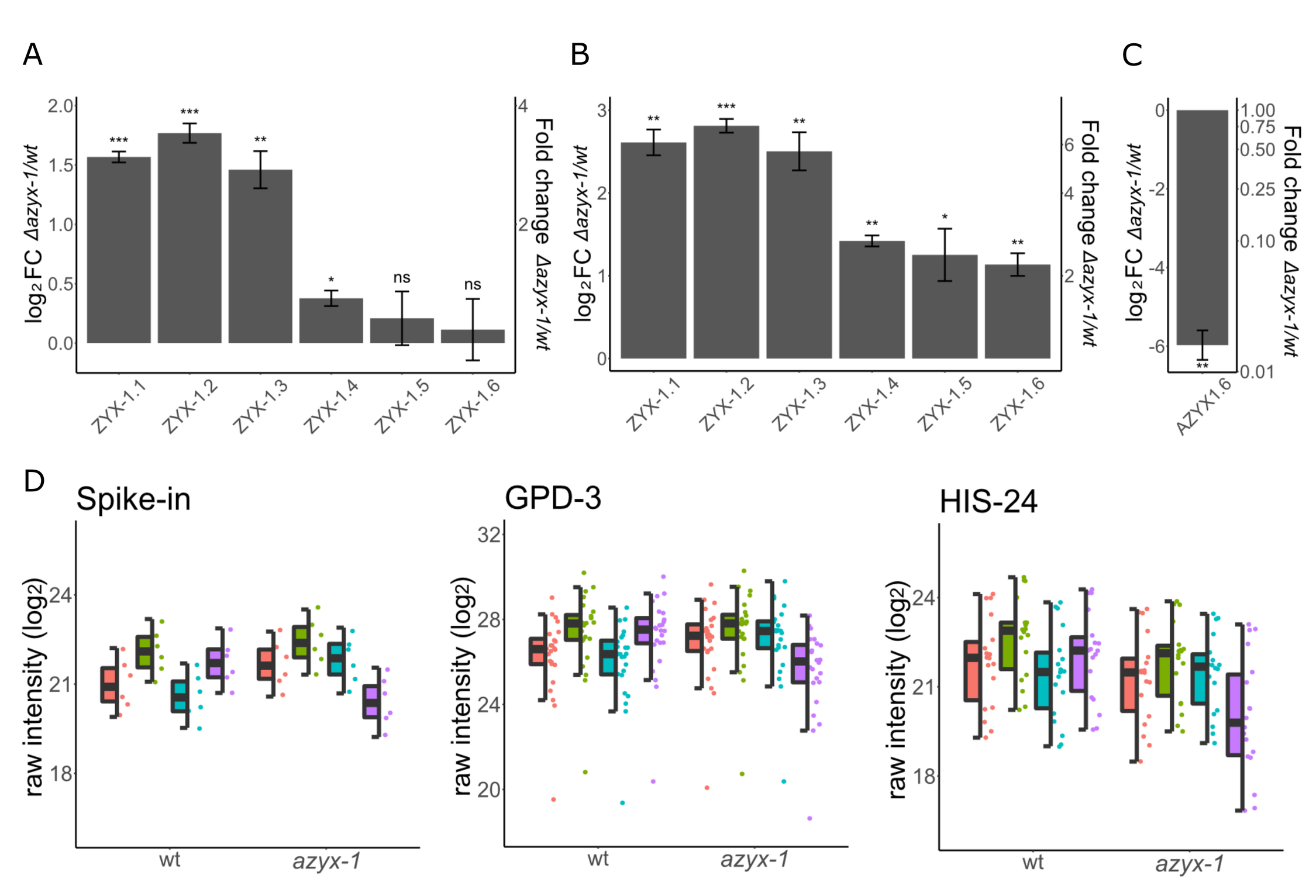

Supplement: S3 Fig — Fold change and standard error of individual ZYX-1 peptides in azyx-1 mutant vs. wt with data normalized to (A) spike-in peptide or (B) HIS-24. (C) Fold change and standard error of the only detectable AZYX-1 peptide in azyx-1 mutant strain (LSC1898), likely corresponding to AZYX-1b isoform, upon AZYX-1a start codon deletion with a significant down-regulation in comparison to wt at day 1 of adulthood (normalized to GPD-3, p = 0.0017). (D) Distribution of raw intensity of transition ions with mean and standard error for spike-in (1 peptide), GPD-3 (4 peptides), and HIS-24 (3 peptides) across 4 (colored) biological replicates. None of the normalization methods differed significantly from the others (Levene’s test p = 0.991 for normalized ratios of raw intensities, with pairwise p-values vs. spike-in 0.99, vs. GPD-3 0.72, and vs. HIS-24 0.72). Data used to generate figures can be found in S1 Data. (TIF) [file pbio.3002300.s003.tif]

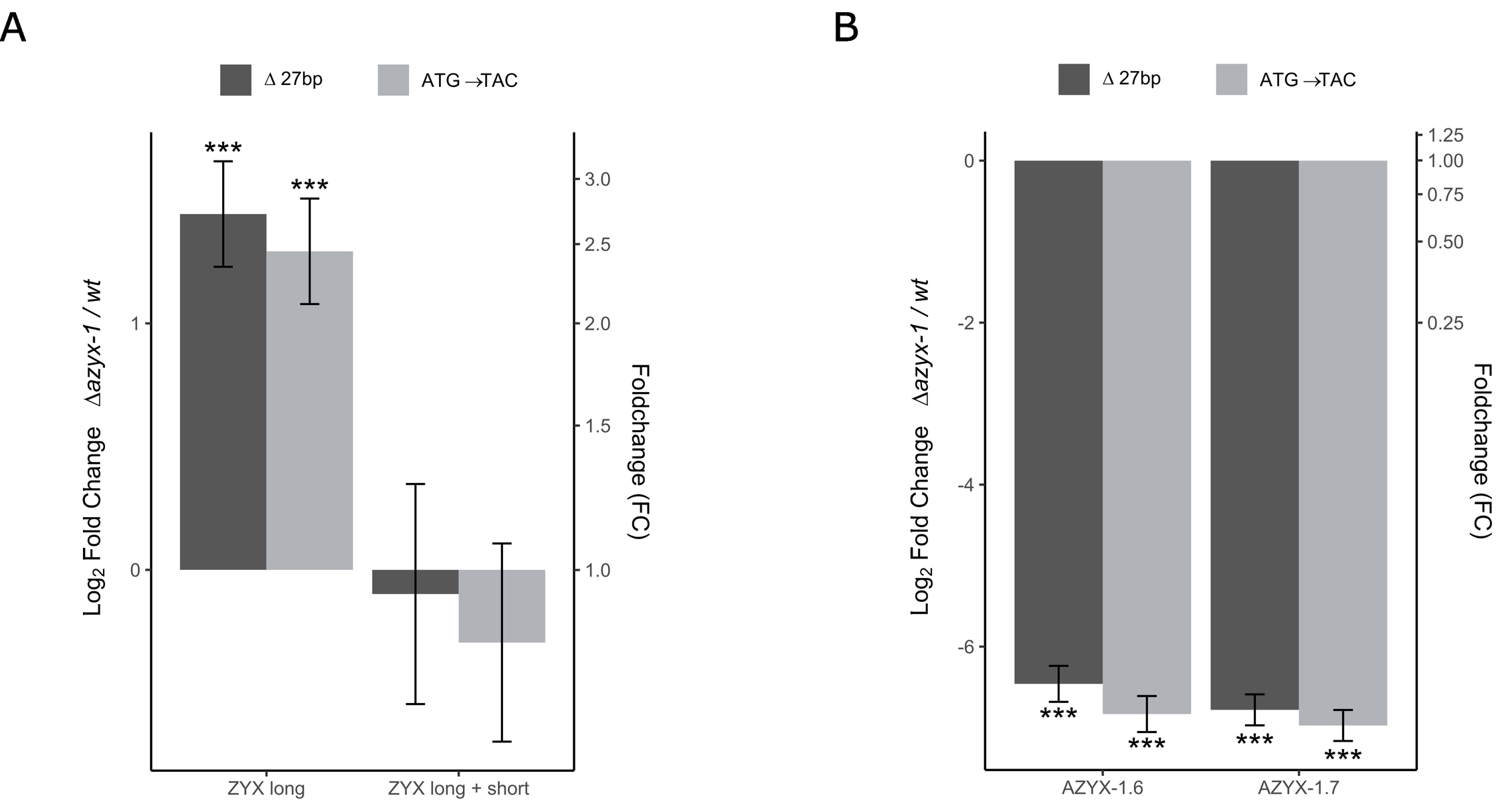

Supplement: S4 Fig — Fold change and standard error of peptides in azyx-1 mutant strains (Δ27bp and ATG -> TAC) corresponding to (A) ZYX-1, where ZYX-1.1–1.3 peptides are specific to long zyxin isoforms and ZYX-1.5 being the only detectable peptide shared between all isoforms, and (B) the only detectable AZYX-1 peptides likely corresponding to AZYX-1b isoform; p-value: *** <0.001, n = 5 biological replicates, L4 larval stage, with data normalized to GPD-3. Data used to generate figures can be found in S1 Data. (TIF) [file pbio.3002300.s004.tif]

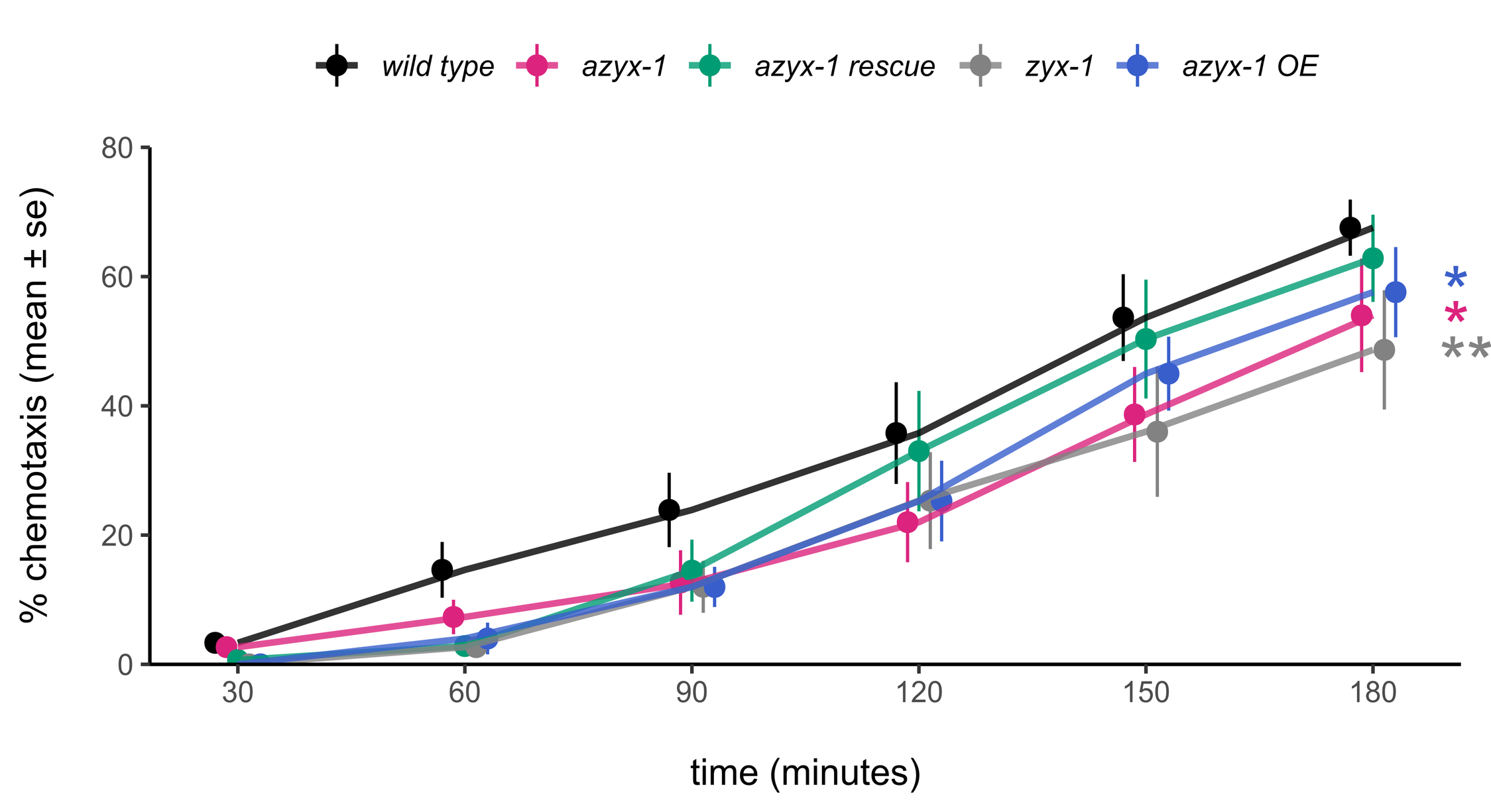

Supplement: S5 Fig — Chemotaxis index of burrowing assay in day 1 adults, as cumulatively observed over 180 min for the azyx-1 (Δ27bp) mutant (LSC1898), rescue (LSC1951), and overexpressor (LSC1950) strains in comparison to positive (zyx-1(gk190)) and negative (wild type) controls, n = 30 per condition in 5 biologically independent replicates (30 × 5); two-way ANOVA p = 0.0025 for strain and time. Tukey HSD wild type vs. azyx-1 OE p = 0.047, vs. azyx-1 (Δ27bp) p = 0.018, vs. zyx-1 p = 0.002. Data used to generate figure can be found in S1 Data. (TIF) [file pbio.3002300.s005.tif]
