# Supplementary material for: azyx-1 is a new gene that overlaps with zyxin and affects its translation in C. elegans, impacting muscular integrity and locomotion
Source: PLoS Biol. 2023 Sep 15;21(9):e3002300. doi: 10.1371/journal.pbio.3002300 (PMC10575671; doi:10.1371/journal.pbio.3002300)
Supplement: Attachment — Submitted filename: 128810_1_rebuttal_2458794_rhy3fm.pdf [file pbio.3002300.s011.pdf]

Revision Plan

Manuscript number: RC-2022-01589R
Corresponding author(s): Temmerman, Liesbet

1. General Statements [optional]

The newly identified *azyx-1* ORF was named *peu-1* in the initial submission of this manuscript, a name that was under consideration with WormBase, who supervise nomenclature of *C. elegans* genes. In consultation with WormBase, the locus was named *azyx-1* instead (the final decision being "*azyx-1* will be attributed to F42G4.11. It will be released in WS287 at the beginning of 2023"). We updated this nomenclature in our submission files, including in reviewer comments pasted below. Please note that other than this, no changes whatsoever were made to the reviewer comments.

2. Description of the planned revisions

REV #3: Specific thoughts for consideration:
Figure 5, Moderate is really minor/moderate with other metrics, and severe is definitely moderate with other metrics. Thus, I'm not sure if normal vs. moderate is needed. This really is a minor point as it doesn't impact results/overall story/importance.
This was also pointed out by reviewer #1. We will rename classification more mildly so.
REV #1 Fig. 5 Even the 'severe' muscle disruption is quite mild (say, in comparison to loss of talin). Perhaps rephrase these categories? The moderate and severe categories also do not look different to me. Show what the muscle cells look like in zyx-1 deletion and overexpression animals. Is there a way to use quantitative imaging to score these? Can azyx-1 phenotypes be rescued or enhanced by expression (or RNAi) of zyxin in the muscle? Also, clarify what age animals are being tested in the muscle and burrowing assay.
We agree and will rename the classes in milder terms. Qualitative scoring (which was done blinded) is the standard in the field as was done according to Dhondt et al. (2021 Dis Model Mech). When tested for muscle integrity and burrowing capacity, animals were day 1 adults. This is mentioned in the Methods section of the current manuscript and will also be included in the captions of the revised figures.
REV #2: I am not convinced by the data presented in Figure 5. There does not seem to be much to distinguish the five genotypes, but I concede that I am not used to looking at this type of data. But why was the muscle phenotype not also examined in the azyx-1 rescue lines?
Because other reviewers that are familiar with these data point out that the observed differences of panels A-B are indeed milder that what is usually seen, we will rename classifications in the manuscript (see responses above). Because the azyx-1 deletion mutant does not differ from controls in the muscle phenotype, there is no phenotype to rescue for this readout, and no rescue strains were generated.
We are not sure what the reviewer may struggle with in (assumedly) panel C (~'to distinguish the five genotypes'). The positive control (zyx-1) behaves as expected in the burrowing assay, with our own mutants within that range, also as expected. All data were scored blinded to avoid any bias and statistical analysis supports the interpretations, all granting confidence to the observed

differences. However, because reviewer#3 also would prefer another representation of the data shown in this panel (see below), we will provide an updated panel representation in the revised manuscript.

REV #3: Figure 5C- Hard to read. Would displaying lines/tragectories make it easier to understand? Would displaying as violin plots for each timepoint/condition make it easier to visualize? Basically in black and white and in color this is hard to visually process.

We will work on another representation for the revised manuscript, since reviewer2 also seemed to struggle with this panel representation.

REV #1: Fig. S2 Match font sizes on Y-axes. Also, indicate any statistical differences and statistics used.

Figure adjustments will be implemented in the revised manuscript as requested.

REV#1: Fig. S3 C, indicate any statistical differences and statistics used.

Figure adjustments will be implemented in the revised manuscript as requested.

REV #2: I am not convinced by the "overexpression" experiments. These are not well controlled, since no evidence is presented that AZYX-1 is being overexpressed in these lines. Also, since we know that extrachromosomal transgenic lines are highly variable, one would need to test the effect of several independent lines to ensure that the effects that the authors observe are indeed associated with AZYX-1 overexpression and not simply an idiosyncratic effect of the genetic background of a given strain. Finally, there does not seem to be an obvious mechanism by which overexpression of AZYX-1 can impact ZYX-1 function. That doesn't rule out an effect, but based on the data as it is, it is premature to propose such a mechanism. The authors need to show that multiple overexpression lines do reproducibly overexpress AZYX-1 and that this results in reproducible effects of zyx-1 phenotypes.

The extrachromosomal strains are indeed variable, but because the background is wild type (in contrast to a deletion mutant background for rescue strains), an overdose of the target provided is expected. As requested in the cross-consultation reviewer communication, we will include quantitative data in our revised manuscript that shows that the used strains (LSC1950, LSC1960, LSC2000) indeed are overexpressors.

REV #2: The data presented in Figure 4F needs to be quantified using the same format as was presented in Figure 4B.

Due to the different genetic background of the strains, this is not possible in the exact same way (the red signal of LSC1998 & LSC1999 is not unique to zyxin). We understand that in essence, the reviewer would like us to include a more quantitative representation of these data, and will update the figure accordingly.

REV #2: What is the difference between the overexpression transgenic lines and the "rescuing" transgenic lines? In the Materials and Methods, the same concentration of plasmid was used in injections - so these likely give the same approximate level of transgenic expression.

The genetic background: a rescue line adds wt DNA back to a mutant background, while in an OE strain it is added into a wt background. While this can already be derived from the genotype

details in Supplemental Table S1, we apologize for not specifying this in the methods section, as it is common practice in the field. These specifications will be added to the revised manuscript.

REV #2: I am not clear what features are being used to characterise the myofibril structures into the three categories. Can the authors annotate the images to indicate the diagnostic features?
The reviewer is correct that manual classification is rather poorly defined in general, which is why it is scored blinded (here as per Cothren et al., 2018 Bio Protoc). We adhered to the reference images by Dhondt et al. (2021, Dis Mod Mech) with visual assessment based on how tightly organized (~parallel) myofilaments are organized, assessing overall increases of bends or breaks in individual myofibers as leading to a less aligned pattern (cf. Fig. 1 of Dhondt et al.). We will add this information more explicitly to the Methods section of the revised manuscript.

3. Description of the revisions that have already been incorporated in the transferred manuscript

REV #1: Fig. 4 would be better if the control (A) and azyx-1OE (B) worms were more similar in age and size
The panels of this figure were not to the exact same scale, we apologize if the reviewer found this confusing. We have rescaled the panels so that this is less confusing. The animals are all day 1 adults.

REV #1: Abstract: Clarify what is meant by 'putative syntenic conservation' or rephrase, simply stating that the existence of an ORF overlapping with the 5' region of zyxin is conserved
This has been rephrased according to request.

REV #1: Line 24: Clarify these are synthetic phenotypes (not caused by loss of zyx-1/azyx-1 alone). Loss of zyx-1 alone results in very mild phenotypes.
While the original sentence already pointed this out, we rephrased the text to make clear that these observations require the dystrophic mutant background.

REV #1: Line 28: Start new paragraph
The new paragraph was started a sentence earlier, according to rev#2 request.

REV #1: Line 31: Not clear what is meant by 'post-transcriptional regulation can be further propagated'- maybe reword to 'alternative and overlapping open reading frames (ORFs) arising from polycistronic mRNA can regulate translation' or something simpler like that.
This has been rephrased according to request.

Revision Plan

REV #1: Line 56-57: Is this because most C. elegans transcripts start with the splice leader SL1 or SL2 rather than the adjacent 5' sequence? Is that relevant for zyx-1? Recommend commenting briefly on this.

We did not look into this for all possible u(o)ORFs in C. elegans, which also is not the focus of the manuscript, so we cannot make general statements. As part of the annotation procedure of azyx-1, WormBase verified that indeed several pieces of evidence, including available phyloCSF data for exon 1, SL1s, RNASeq and Nanopore data, all support its annotation, as well as its translation from the zyx-1 long transcripts (albeit with different start and in different reading frame).

REV #1: Line 78: Delete the word 'other'

Done

REV #1: Line 122: zyx-1

Done

REV #1: Line 137: 'lead' should be 'led'

Done

REV #1: Line 158: rephrase 'only the long ones' to indicate which isoforms more precisely

Done (these are a/e, cf. Luo et al. 2014, Development)

REV #1: Line 195: Rephrase. Unclear what is meant by 'highlights the evasiveness of non-canonical ORFs from functional annotation'

Done; this was rephrased to "This exemplifies how non-canonical ORFs can escape functional annotation, …".

REV #1: Various locations: I think it will be more clear to the reader to consistently refer to the burrowing assay as 'burrowing assay' rather than chemotaxis. I recommend adding a brief description of the burrowing assay to the results section.

Wording has been updated, we can provide a short context sentence to the results section of the revised manuscript.

REV #2: I'm not sure how to interpret the significance of the u/ouORFs across short and large phylogenetic distances. One would presume that there might not be primary amino acid conservation if the regulation simply takes by interference with ribosome scanning and translocation. Here some statistical analysis would help with assessing the significance of these observations. How unusual is it to find u/uoORFs in the 5' UTRs of gene encoding zyxin family members versus in general for the species analysed?

This is indeed the very question we are asking in the manuscript, and there is a clear reason why we refrain from making significance statements. At the moment, all relevant available metadata are used for the analysis in the manuscript, leading to the communication of the synteny-related findings as they are currently presented. This is due to the dependency on translatomics data to find credible u(o)ORFs, and there aren't very many translatomics datasets available, only for a limited set of species so far. Our manuscript contains all relevant OpenProt data, which are derived from only 9 animal species so far. As shown in Table S4, 14 zyxin orthologs belonging to 7 species have associated u(o)ORFS, for two species only overlapping ORFs are present in the database. While more and more datasets will undoubtedly become available in the next years, the findings in the manuscript are as complete as currently possible: we do find evidence of

u(o)ORFs associated with zyxin orthologs in these species, some of which are evolutionarily distantly related to C. elegans.

REV #2: The authors state that there is evidence for synteny and coding region conservation. The data supporting this assertion is not well presented. Presentation and analysis of multiple sequence alignments of the putative homologues involved would strengthen the assertion of synteny considerably.
We apologize if the reviewer misunderstood: we discuss likely syntenic conservation, not coding region conservation. The latter is not mentioned in our manuscript, and in fact not convincing indeed. This is not surprising given the bigger sequence diversity observed at the N terminus of zyxins and the partial overlap of these coding sequences, and in line with observations of several others in the RiboSeq community that many identified uORFs are conserved between orthologous genes, but poorly conserved at the amino acid level (e.g. community-driven communication by Mudge et al., BioRxiv 2021 and references therein).

REV #2: The authors are oddly coy about the molecular details of the 27 bp deletion used to study the loss of azyx-1 function. In the absence of these details, it is not possible to assess the validity of these experiments. We need to be given the full molecular details of the allele - precisely which nucleotides are deleted? And how do they affect the coding regions of zyx-1 and azyx-1?
I am also confused about why the authors made a deletion allele rather than mutating the AUG of AZYX-1? This would be a cleaner experiment to interpret. Based on the data presented, there are two possible interpretations in addition to the one suggested by the authors: 1) the 27 bp deletion impacts zyx-1 expression due to its impact on the zyx-1 coding region (the coding regions of azyx-1 and zyx-1 overlap); 2) the deletion mutation deletes critical transcriptional control elements. A simpler mutation of the azyx-1 AUG via CRISPR might allow them to rule out the possibility that they have simply compromised a transcriptional control element or damaged the coding region of ZYX-1.
As mentioned above and as will be included more clearly so in the revised manuscript: the deletion is 182-155bp (27bp) upstream of the zyx-1a start site. This was a mutant that could easily be generated via CRISPR, so we proceeded with this one. This edit rules out option1 (there is no change of the zyxin coding region), but (as also considered but addressed differently in the manuscript; see below) retains alternative interpretation 2. There are no regulatory regions or transcription factor binding sites known for the (a)zyx-1 locus (verified in current WormBase version WS285), but that does certainly not fully rule out the possibility either. Rather than creating a series of azyx-1 mutants, be they SNP or small deletion mutants, that would suffer from the exact same duality in possible interpretation, we chose to combine the deletion mutant with rescue and overexpression strains. Because these latter strains do not affect the endogenous zyxin regulatory region, they add far more credibility to the interpretation, than alternative mutants in the azyx-1/zyx-1 locus would.

REV#2. The narrative flow of the introduction could be improved by the judicious use of paragraphs. Line 12, for instance is a clear paragraph break, as is line 24.
done

REV #3: Specific thoughts for consideration:
3) Could more be said about overlapping genes/regulation in humans? Again, not critical _but_ this is such a great piece of work that it would be useful to guide human subjects researchers as to how to best further your work.
It is unclear whether the reviewer would like to see an extended introduction and/or discussion. We tried to meet this request without drifting too much from the focus of our current communication by adding the following to the introduction (lines 41-47 of the current draft): "From a more human-centred future perspective, uORFs are a rather unexplored niche for translational research: with a predicted prevalence in over 50% of human genes and first examples regulating translation of disease-associated genes already emerging (Lee et al. 2021; Schulz et al. 2018), the field is bound to not only lead to more fundamental, but also application-oriented insights. Keeping this broader context in mind, we here focus on more fundamental principles of uORFs in a model organism context.".

4. Description of analyses that authors prefer not to carry out

REV#1: Does azyx-1 have zyx-1-independent functions or other regulatory targets?
This is an interesting question that is not yet addressed. While this is possible, it is beyond the scope of our current communication. Since the reviewer does not request anything concrete, we would prefer to leave this for follow-up research. While this notion is included in the manuscript, we are happy to more explicitly address this question in the discussion as well.

REV#1: Do the burrowing assay results reflect a neuronal or a muscle function for AZYX-1? Or both?
Our manuscript indeed does not yet delve into tissue-specific actions of this newly discovered ORF. While interesting, and in line with reviewer #3's remark, this would be valuable for follow-up research, but is beyond the scope of our current communication. We will make sure the concept is clearly mentioned in the discussion of our findings.
REV #3: Specific thoughts for consideration:
1) Could more be done/said about neruo vs, muscular effects of azyx-1 and zyx-1. I appreciate this is beyond the scope of the present manuscript and therefore does not require response if you don't have data or it makes telling the story you want to tell more difficult.
We agree with the reviewer that spatially resolving some of these observations would be a next interesting step, which indeed is beyond the scope of our current communication.

REV#1: Fig. 2A very faint, increase brightness/contrast?
We did not adjust brightness or contrast for any of the figures, an no such requests were made by other reviewers. We greatly prefer presenting the data as unedited as possible, and would like to request the journal's preference for action here.

5. Remaining reviewer comments & responses not highlighted above

CROSS-CONSULTATION COMMENTS

The following is a conversation among the three referees:

REFEREE #2: I appear to be the dissenting voice in terms of concern about the details of the 27 bp deletion and the "overexpression" constructs. I would be interested to know your opinions regarding my comments on these issues.

REFEREE #1: I think adding the details of the 27 bp deletion is a reasonable request. It is probably not possible to disambiguate entirely the two effects of the deletion, and changing the start codon may result in an alternate start with other downstream effects. I think just explaining it more fully in the methods would satisfy my concerns.

REFEREE #2: What about the issues with the overexpresssion? In my experience, presence of multicopy transgenes on an extrachromosomal arrays might not lead to over expression of the gene involved? This needs to be verified in some way.

REFEREE #1: You are right about that. If the construct is tagged in some way they could try a western. I would recommend they integrate the transgenes, or just show results from several lines as you suggest.

REFEREE #3: I agree the 27 bp deletion and over expression are reasonable technical issues. However, I view this a techical details vs. critical details for the novel regulatory mechanism. The point about ability to judge conservation is also reasonable but until the theory is firmly out there it is hard to test the conservation and broader applicability to other genes/proteins. Thus, while asking for additional information on these issues is reasonable I do not see the inability to address beyond highlighting as limitation in the text as critical to the overall validity of the work.

REFEREE #2: I disagree with Reviewer #3, without knowing the details of 27 bp deletion the most reasonable interpretation of the data is simply that it is a loss-of-function allele of zyx-1. This goes beyond "technical" - at present there is no unequivocal evidence that azyx-1 has any functional significance beyond that it is expressed as a peptide.

REFEREE #3: I've been back through the manuscript. They have sequenced the deletion and therefore should be able to provide that information to satisfy the issue(s). For the over expression, short of silencing my experience is that they do over express and when you have multiple lines some express more than others (and some silence more than others). If you want evidence that the peptide is over expressed ask them to quantify via mass spec if it isn't tagged and they can't do a Western. Clearly they have work leading expertise in quantitative mass spec proteomics in C. elegans and should be able to do that. Generally speaking, rescue of a deletion is a pretty good sign that the expression is working though (and is an accepted standard).

We apologize if this was not clear from the manuscript, and will clearly include the details in the Methods section: the deletion is 182-155bp (27bp) upstream of the zyx-1a start site, at AT|G+26|TTC. This was confirmed by sequencing; the oligos used for this are listed in table S3 of the manuscript.

We address the confusion of rescue and overexpression above, in response to reviewer #2 (who echoes this confusion here).

Revision Plan

Reviewer #1 (Evidence, reproducibility and clarity (Required)):

This is a very interesting paper about a gene regulatory mechanism in a type of poly-cistronic mRNA in which alternate starts/open reading frames lead to production of two different proteins from the same locus. AZYX-1 is a predicted 166 aa protein, translated from the 5'UTR of zyx-1. Two isoforms are expressed from the 5' UTR and coding region of zyx-1. The presence of overlapping transcripts with zyxin orthologs appears to be conserved in other animals. The authors provide spectroscopic evidence AZYX-1 is indeed translated, and show AZYX-1 can regulate zyx-1 expression. Intriguingly, it seems azyx-1 inhibits zyx-1 expression in cis (deletion of azyx-1 increases ZYX-1 peptides), but AZYX-1 promotes zyx-1 expression in trans (overexpression of AZYX-1 increases ZYX-1 expression).

Reviewer #1 (Significance (Required)):

Nature and significance of the advance: This is a very interesting paper about a gene regulatory mechanism in a type of poly-cistronic mRNA encoding azyx-1 and zyx-1. Intriguingly, it seems azyx-1 inhibits zyx-1 expression in cis (deletion of azyx-1 increases ZYX-1 peptides), but AZYX-1 promotes zyx-1 expression in trans (overexpression of AZYX-1 increases ZYX-1 expression).
Compare to existing published knowledge: This is the first study of its type on zyx-1.
Audience: Those interested in gene regulatory mechanisms and in zyxin.
My expertise: C. elegans cytoskeleton, cell migration, acto-myosin contractility.

Reviewer #2 (Evidence, reproducibility and clarity (Required)):

Summary:
The authors build on previous work defining upstream and upstream-overlapping open reading frames (uORF and uoORFs, respectively) by focussing on a specific locus azyx-1, which the authors propose influences the expression of the gene encoding the sole zyxin family in C. elegans, zyx-1. They present evidence suggestive of u/uoORFs being a common feature of zyxin family genes in other animals, hinting that perhaps this is a conserved mechanism of gene expression regulation for these genes. In which case, studies in C. elegans would be valuable to elucidate the mechanism involved.
Using a fluorescent reporter strategy, they show that azyx-1 is expressed in the same tissues as zyx-1, which is to be expected since their share the same transcriptional control elements.
They also characterise the peptide steady state levels of both ZYX-1 and AZYX-1 isoforms, suggesting that while overall ZYX-1 levels decline with age, those for AZYX-1 are generally maintained. The significance of these observations was not immediately obvious to me - a priori it is difficult to assess what relative wild type steady-state levels one might expect if AZYX-1 translation impacted ZYX-1 expression.
The authors propose that expression of AZYX-1 leads to inhibition of ZYX-1 translation through the standard model by which u/uoORFs impact translation of downstream ORFs. To test this, they generated a 27 bp deletion "at the beginning of the azyx-1 ORF". This deletion clearly correlated with a reduction in ZYX-1 expression.
Finally, the authors generated lines designed to overexpress AZYX-1, testing the hypothesis

that AZYX-1 might influence ZYX-1 in trans. Though here, it is not obvious by what mechanism this might operate, and the effect-sizes involved are modest.

Reviewer #2 (Significance (Required)):

The authors propose an interesting interaction between an important regulator of cellular behaviour (zyxin) and the u/uoORF that potentially regulates its expression - if validated by further experimentation, this would add to the growing evidence for the importance of the 5' UTR as a source of gene regulatory activity. Such regulation is well described in yeast, but there are fewer examples in animals, particularly in genetically tractable systems such as C. elegans. The work would primarily be of interest to researchers interested in understanding the spectrum of such activity in C. elegans. My own area of expertise, RNA-splicing and the post-transcriptional regulation of C. elegans gene expression, is not directly related to the research presented in the manuscript, but I am familiar with the general concepts and developments involved.

Reviewer #3 (Evidence, reproducibility and clarity (Required)):

Summary:

The authors find that azyx-1 is a non-cononical gene with overlapping genomic localization to the gene zyx-1 in C. elegans. The authors also find preliminary evidence that similar genes with overlapping localization to zyxin genes exist in other species. The authors provide evidence for the tissue specific distribution of azyx-1 expression. The authors further provide evidence for azyx-1 and zyx-1 expression with age. Importantly, these data demonstrate differences in azyx-1 and zyx-1 protein products biological importance/relevance as they display differences with age. The authors provide evidence that azyx-1 expression influences zyx-1 expression in multiple ways. Lastly, the authors demonstrate that azyx-1 expression influences muscle structure and neuromuscular function. The authors use a combination of bioinformatic, protein biochemistry, genetic/transgenic, histologic, and physiologic methods to make these points. With regards to methods, the range/breadth is impressive and appropriate. In many ways the manuscript it is a tour de force in modern molecular biology with a focus on translational medicine. With regards to species, the in vivo experiments are solely C. elegans but the computational data include Fly, Bull, and Mouse.

The key conclusions are convincing. There are no major claims that require qualification as preliminary or speculative. No additional experiments are essential to support the claims of the paper. The data and methods are presented in such a way that they can be reproduced. The experiments are adequately replicated and the statistical analysis is adequate.
Prior studies are references appropriately. The text and figures are mostly clear and accurate.
We would like to thank the reviewer for their appreciation of our efforts and research approach.

Reviewer #3 (Significance (Required)):

Conceptually this is a massive/ground breaking piece of work. Essentially, the authors are demonstrating a novel mechanism of regulation of gene/protein expression that, really, hasn't

been reported before. What is particularly notable is that it appears, unsurprisingly, as correctly stated by the authors, to be evolutionarily conserved and not well reported in the literature. As with many classical molecular biology papers, and the more recent (e.g. RNAi, lncRNA) genetic papers, this manuscript hold the promise of transforming biology/medicine. The range of methods employed and the linking of molecular biology to pathophysiology was impressive. The audience that will be interested in this work includes: geneticists, proteomics researchers, evolutionary researchers, molecular biologists, physiologists, ageing researchers, muscle researchers, and muscle disease researchers. Thus, the interested audience is broad. My field of expertise with regards to this manuscript is: C. elegans, Mass Spec, Proteomics, genomic regulation, genetics, transgenics, histology, muscle, and physiology. There are no parts of this manuscript that I do not feel I have insufficient expertise to evaluate. I congratulate the authors on a highly significant, cross disciplinary, manuscript, that should impact multiple sub-areas of biology.
