# Supplementary material for: azyx-1 is a new gene that overlaps with zyxin and affects its translation in C. elegans, impacting muscular integrity and locomotion
Source: PLoS Biol. 2023 Sep 15;21(9):e3002300. doi: 10.1371/journal.pbio.3002300 (PMC10575671; doi:10.1371/journal.pbio.3002300)
Supplement: Attachment — Submitted filename: response to reviewers.PDF [file pbio.3002300.s014.PDF]

There were no reviewer comments to address. Please find a short confirmation of having addressed the editorial and journal comments below. As requested by the editor, this information is also present in the cover letter:

- We updated the title as per your suggestion, including only a minor change (replacing "thereby" with a comma ","). The title of the revised manuscript now reads "*azyx-1* is a new gene that overlaps with *zyxin* and affects its translation in *C. elegans*, impacting muscular integrity and locomotion".
- In line with PLOS Data Policy, all numerical data belonging to main and supplemental figures ("Figure 3A-D, 4B-D, 4G, 5B-C, S2A-B, S3A-D, S4A-B, S5") are referred to in the figure legends and are available in supplemental file "S1_Data.xlsx".
- Apologies for the confusion regarding the deposited mass spectrometry data. We verified that all raw MS data are accessible via the Panorama Public link (https://panoramaweb.org/azyx1.url) and removed the unneeded reference to ProteomeXchange.
- All relevant figure legends include information on underlying data (S1_Data.xlsx) and all supplemental data files have legends.
- The Data Statement in the submission system has been updated and correctly describes where all data can be found.
- There is no need for changes to the reference list; it is indeed complete and correct.
